# Supplementary material for: Current status of cervical cytology during pregnancy in Japan
Source: PLoS One. 2021 Jan 7;16(1):e0245282. doi: 10.1371/journal.pone.0245282 (PMC7790376; doi:10.1371/journal.pone.0245282)
Supplement: Attachment — Submitted filename: Revised letter.doc [file pone.0245282.s002.doc]

December 23, 2020

Editorial office

PLOS ONE

Dear Editors,

We would like to thank you and the reviewer for the comments and critique of our manuscript entitled ‘Current status of (uterine) cervical cytology during pregnancy in Japan’. We have been able to respond positively to each comment and we believe the paper has been strengthened. The changes are highlighted as red colored text.

The protocol for this study was approved by the Ethics Committee of the Japan Association of Obstetricians and Gynecologists (JAOG). Because no individual can be identified under the protocol of this retrospective study of medical records, the ethics committee waived the requirement for informed consent with each subject. In addition, we have confirmed that all data were fully anonymized before we accessed them.

The authors received no specific funding for this work.

Therefore,

1. We have re-confirmed PLOS ONE style.
2. We have added the comments concerning consent in the Methods.
3. There are no financial disclosure.
4. There are no COI.

In addition, the data are published in Figshare (10.6084/m9.figshare.13347299, Title: Current status of cervical cytology in Japan).

Responses to the Editor

Many thanks for your careful reading of the manuscript. We appreciate your comments very much. Thank you very much for your suggestions. We have re-written the manuscript heavily, relying on your suggestions.

- We have changed from CCC to conventional cervical cytology. Otherwise, we have used the word of Pap smear as appropriate. We have changed to ‘prevalence’, ‘cervical cytology, and ‘teenagers’.
- Results/Abstract: Thank you very much for your suggestions. We have corrected the sentences/words according to your suggestions.
- Discussion/Abstract: We have corrected the sentences/words according to your suggestions. We have re-learned the recommendations from European specialists based on the literatures you suggested (Ref: 28,37,38). We have reflected on our own statements in the first revised manuscript. We have re-written the Discussion & Conclusions with the European recommendations. In addition, we have explained the HPV vaccine crisis in the Introduction

Responses to Reviewer 1,

Many thanks for your careful reading of the manuscript. We appreciate your comments very much. Thank you very much for your suggestions. We have re-written the manuscript heavily, relying on your suggestions.

1. Thank you very much for your suggestion in the Discussion. With your suggestion, we realized the need for a detailed examination of the results of teenage women. We have added the comments concerning the Japanese Government recommendation (a), examination methods (b) and examination rate of HPV testing for teenage women (c).
2. In the Introduction, we have re-written to add the comments of HPV vaccine crisis in Japan. In addition, we have changed the Introduction and Methods as suggested.
3. Thank you very much for your corrections. The manuscript has been re-checked by an English native speaker.

Responses to Reviewer 2,

Many thanks for your careful reading of the manuscript. We appreciate your comments very much. Thank you very much for your suggestions. We have re-written the manuscript heavily, relying on your suggestions.

1. We have added some references to add the comments of vaccine crisis in Japan.
2. The manuscript has been re-checked by an English native speaker.
3. We have changed the Introduction and Methods as suggested.
4. We have added the introduction and comments of tables.
5. We have separated the Discussion to 2 parts. We have added the Conclusion.

We do hope and trust that with these changes the manuscript is now acceptable for publication.

Thank you very much, again.

Sincerely yours,
Shunji Suzuki, MD
Department of Obstetrics and Gynecology,
Japanese Red Cross Katsushika Maternity Hospital
5-11-12-2 Tateishi, Katsushika-ku, Tokyo 124-0012 Japan
Tel: +81-3-3693-5211
Fax: +81-3-3694-8725
e-mail: czg83542@mopera.ne.jp
